# Supplementary material for: Resolving the full spectrum of human genome variation using Linked-Reads
Source: Genome Res. 2019 Apr;29(4):635–45. doi: 10.1101/gr.234443.118 (PMC6442396; doi:10.1101/gr.234443.118)
Supplement: Supplemental Material [file supp_gr.234443.118_Supplemental_longranger-2.2.zip › longranger-2.2.tar.bz2!/lr2.2-public/lib/go/src/code.google.com/vitess/docs/overview/index.html]

Vitess / Understanding Vitess 


Toggle navigation

- Overview
- Guides
- Reference
- Blog
- GitHub

- #### Overview

  - What is Vitess
  - Scaling MySQL with Vitess
  - Key Concepts
- #### Getting Started

  - Run Vitess on Kubernetes
    - Custom Docker Build
  - Run Vitess Locally
- #### User Guide

  - Introduction- Client Libraries- Backing Up Data- Reparenting
        - Schema Management
        - VSchema Guide
        - Schema Swap (Tutorial)
        - Sharding
          - Horizontal Sharding (Tutorial, automated)
          - Horizontal Sharding (Tutorial, manual)
          - Sharding in Kubernetes (Tutorial, automated)
          - Sharding in Kubernetes (Tutorial, manual)
        - Vitess Sequences
        - MySQL Server Protocol
        - Vitess and Replication
        - Update Stream
        - Row Based Replication
        - Topology Service
        - Transport Security Model
        - Launching
          - Scalability Philosophy
          - Production Planning
          - Server Configuration
          - 2PC Guide
          - Troubleshooting
        - Upgrading
- #### Reference Guides

  - Vitess API- vtctl Commands
- #### Other Resources

  - Presentations- Blog- Roadmap
- #### Contributing

  - Contributing to Vitess- GitHub Workflow- Code Reviews
- GitHub

# Understanding Vitess

- #### Overview

  - What is Vitess
  - Scaling MySQL with Vitess
  - Key Concepts
- #### Getting Started

  - Run Vitess on Kubernetes
    - Custom Docker Build
  - Run Vitess Locally
- #### User Guide

  - Introduction- Client Libraries- Backing Up Data- Reparenting
        - Schema Management
        - VSchema Guide
        - Schema Swap (Tutorial)
        - Sharding
          - Horizontal Sharding (Tutorial, automated)
          - Horizontal Sharding (Tutorial, manual)
          - Sharding in Kubernetes (Tutorial, automated)
          - Sharding in Kubernetes (Tutorial, manual)
        - Vitess Sequences
        - MySQL Server Protocol
        - Vitess and Replication
        - Update Stream
        - Row Based Replication
        - Topology Service
        - Transport Security Model
        - Launching
          - Scalability Philosophy
          - Production Planning
          - Server Configuration
          - 2PC Guide
          - Troubleshooting
        - Upgrading
- #### Reference Guides

  - Vitess API- vtctl Commands
- #### Other Resources

  - Presentations- Blog- Roadmap
- #### Contributing

  - Contributing to Vitess- GitHub Workflow- Code Reviews

Vitess is a database solution for scaling MySQL. It's architected to run as
effectively in a public or private cloud architecture as it does on dedicated
hardware. It combines and extends many important MySQL features with the
scalability of a NoSQL database. Vitess has been serving all YouTube database
traffic since 2011.

## Vitess on Kubernetes

Kubernetes is an open-source orchestration system for Docker containers, and Vitess can run as a Kubernetes-aware cloud native distributed database.

Kubernetes handles scheduling onto nodes in a compute cluster, actively manages workloads on those nodes, and groups containers comprising an application for easy management and discovery.
This provides an analogous open-source environment to the way Vitess runs in YouTube,
on the predecessor to Kubernetes.

Quickstart

## Comparisons to other storage options

The following sections compare Vitess to two common alternatives, a vanilla MySQL implementation and a NoSQL implementation.

### Vitess vs. Vanilla MySQL

Vitess improves a vanilla MySQL implementation in several ways:

| Vanilla MySQL | Vitess |
| --- | --- |
| Every MySQL connection has a memory overhead that ranges between 256KB and almost 3MB, depending on which MySQL release you're using. As your user base grows, you need to add RAM to support additional connections, but the RAM does not contribute to faster queries. In addition, there is a significant CPU cost associated with obtaining the connections. | Vitess' gRPC-based protocol creates very lightweight connections. Vitess' connection pooling feature uses Go's concurrency support to map these lightweight connections to a small pool of MySQL connections. As such, Vitess can easily handle thousands of connections. |
| Poorly written queries, such as those that don't set a LIMIT, can negatively impact database performance for all users. | Vitess employs a SQL parser that uses a configurable set of rules to rewrite queries that might hurt database performance. |
| Sharding is a process of partitioning your data to improve scalability and performance. MySQL lacks native sharding support, requiring you to write sharding code and embed sharding logic in your application. | Vitess uses range-based sharding. It supports both horizontal and vertical resharding, completing most data transitions with just a few seconds of read-only downtime. Vitess can even accommodate a custom sharding scheme that you already have in place. |
| A MySQL cluster using replication for availability has a master database and a few replicas. If the master fails, a replica should become the new master. This requires you to manage the database lifecycle and communicate the current system state to your application. | Vitess helps to manage the lifecycle of your database scenarios. It supports and automatically handles various scenarios, including master failover and data backups. |
| A MySQL cluster can have custom database configurations for different workloads, like a master database for writes, fast read-only replicas for web clients, slower read-only replicas for batch jobs, and so forth. If the database has horizontal sharding, the setup is repeated for each shard, and the app needs baked-in logic to know how to find the right database. | Vitess uses a topology backed by a consistent data store, like etcd or ZooKeeper. This means the cluster view is always up-to-date and consistent for different clients. Vitess also provides a proxy that routes queries efficiently to the most appropriate MySQL instance. |

### Vitess vs. NoSQL

If you're considering a NoSQL solution primarily because of concerns about the scalability of MySQL, Vitess might be a more appropriate choice for your application. While NoSQL provides great support for unstructured data, Vitess still offers several benefits not available in NoSQL datastores:

| NoSQL | Vitess |
| --- | --- |
| NoSQL databases do not define relationships between database tables, and only support a subset of the SQL language. | Vitess is not a simple key-value store. It supports complex query semantics such as where clauses, JOINS, aggregation functions, and more. |
| NoSQL datastores do not support transactions. | Vitess supports transactions within a shard. We are also exploring the feasibility of supporting cross-shard transactions using 2PC. |
| NoSQL solutions have custom APIs, leading to custom architectures, applications, and tools. | Vitess adds very little variance to MySQL, a database that most people are already accustomed to working with. |
| NoSQL solutions provide limited support for database indexes compared to MySQL. | Vitess allows you to use all of MySQL's indexing functionality to optimize query performance. |

## Features

- **Performance**

  - Connection pooling - Scale front-end connections while optimizing MySQL performance.
  - Query de-duping – Reuse results of an in-flight query for any identical requests received while the in-flight query was still executing.
  - Transaction manager – Limit number of concurrent transactions and manage deadlines to optimize overall throughput.
- **Protection**

  - Query rewriting and sanitation – Add limits and avoid non-deterministic updates.
  - Query blacklisting – Customize rules to prevent potentially problematic queries from hitting your database.
  - Query killer – Terminate queries that take too long to return data.
  - Table ACLs – Specify access control lists (ACLs) for tables based on the connected user.
- **Monitoring**

  - Performance analysis: Tools let you monitor, diagnose, and analyze your database performance.
  - Query streaming – Use a list of incoming queries to serve OLAP workloads.
  - Update stream – A server streams the list of rows changing in the database, which can be used as a mechanism to propagate changes to other data stores.
- **Topology Management Tools**

  - Master management tools (handles reparenting)
  - Web-based management GUI
  - Designed to work in multiple data centers / regions
- **Sharding**

  - Virtually seamless dynamic re-sharding
  - Vertical and Horizontal sharding support
  - Built-in range-based, or application-defined sharding support

## Architecture

The Vitess platform consists of a number of server processes, command-line utilities, and web-based utilities, backed by a consistent metadata store.

Depending on the current state of your application, you could arrive at a full Vitess implementation through a number of different process flows. For example, if you're building a service from scratch, your first step with Vitess would be to define your database topology. However, if you need to scale your existing database, you'd likely start by deploying a connection proxy.

Vitess tools and servers are designed to help you whether you start with a complete fleet of databases or start small and scale over time. For smaller implementations, vttablet features like connection pooling and query rewriting help you get more from your existing hardware. Vitess' automation tools then provide additional benefits for larger implementations.

The diagram below illustrates Vitess' components:

### Topology

The Topology Service is a metadata store that contains information about running servers, the sharding scheme, and the replication graph. The topology is backed by a consistent data store. You can explore the topology using **vtctl** (command-line) and **vtctld** (web).

In Kubernetes, the data store is etcd. Vitess source code also ships with Apache ZooKeeper support.

### vtgate

**vtgate** is a light proxy server that routes traffic to the correct vttablet(s) and returns consolidated results back to the client. It is the server to which applications send queries. Thus, the client can be very simple since it only needs to be able to find a vtgate instance.

To route queries, vtgate considers the sharding scheme, required latency, and the availability of the tablets and their underlying MySQL instances.

### vttablet

**vttablet** is a proxy server that sits in front of a MySQL database. A Vitess implementation has one vttablet for each MySQL instance.

vttablet performs tasks that attempt to maximize throughput as well as protect MySQL from harmful queries. Its features include connection pooling, query rewriting, and query de-duping. In addition, vttablet executes management tasks that vtctl initiates, and it provides streaming services that are used for filtered replication and data exports.

A lightweight Vitess implementation uses vttablet as a smart connection proxy that serves queries for a single MySQL database. By running vttablet in front of your MySQL database and changing your app to use the Vitess client instead of your MySQL driver, your app benefits from vttablet's connection pooling, query rewriting, and query de-duping features.

### vtctl

**vtctl** is a command-line tool used to administer a Vitess cluster. It allows a human or application to easily interact with a Vitess implementation. Using vtctl, you can identify master and replica databases, create tables, initiate failovers, perform sharding (and resharding) operations, and so forth.

As vtctl performs operations, it updates the lockserver as needed. Other Vitess servers observe those changes and react accordingly. For example, if you use vtctl to fail over to a new master database, vtgate sees the change and directs future write operations to the new master.

### vtctld

**vtctld** is an HTTP server that lets you browse the information stored in the lockserver. It is useful for troubleshooting or for getting a high-level overview of the servers and their current states.

### vtworker

**vtworker** hosts long-running processes. It supports a plugin architecture and offers libraries so that you can easily choose tablets to use. Plugins are available for the following types of jobs:

- **resharding differ** jobs check data integrity during shard splits and joins
- **vertical split differ** jobs check data integrity during vertical splits and joins

vtworker also lets you easily add other validation procedures. You could do in-tablet integrity checks to verify foreign-key-like relationships or cross-shard integrity checks if, for example, an index table in one keyspace references data in another keyspace.

### Other support tools

Vitess also includes the following tools:

- **mysqlctl**: Manage MySQL instances
- **zk**: Command-line ZooKeeper client and explorer
- **zkctl**: Manage ZooKeeper instances

## History

Vitess has been a fundamental component of YouTube infrastructure since 2011.
This section briefly summarizes the sequence of events that led to Vitess'
creation:

1. YouTube's MySQL database reached a point when peak traffic would soon
   exceed the database's serving capacity. To temporarily alleviate the
   problem, YouTube created a master database for write traffic and a
   replica database for read traffic.
2. With demand for cat videos at an all-time high, read-only traffic was
   still high enough to overload the replica database. So YouTube added
   more replicas, again providing a temporary solution.
3. Eventually, write traffic became too high for the master database to
   handle, requiring YouTube to shard data to handle incoming traffic.
   (Sharding would have also become necessary if the overall size of the
   database became too large for a single MySQL instance.)
4. YouTube's application layer was modified so that before executing any
   database operation, the code could identify the right database shard
   to receive that particular query.

Vitess let YouTube remove that logic from the source code, introducing
a proxy between the application and the database to route and manage
database interactions. Since then, YouTube has scaled its user base
by a factor of more than 50, greatly increasing its capacity to serve
pages, process newly uploaded videos, and more. Even more importantly,
Vitess is a platform that continues to scale.

YouTube chose to write Vitess in Go because Go offers a combination of
expressiveness and performance. It is almost as expressive as Python and
very maintainable. However, its performance is in the same range as Java
and close to C++ in certain cases. In addition, the language is extremely
well suited for concurrent programming and has a very high quality
standard library.

### Open Source First

The open source version of Vitess is extremely similar to the version
used at YouTube. While there are some changes that let YouTube take
advantage of Google's infrastructure, the core functionality is the same.
When developing new features, the Vitess team first makes them work in
the Open Source tree. In some cases, the team then writes a plugin
that makes use of Google-specific technology. This approach ensures that
the Open Source version of Vitess maintains the same level of quality as
the internal version.

The vast majority of Vitess development takes place in the open, on GitHub.
As such, Vitess is built with extensibility in mind so that you can adjust
it to the needs of your infrastructure.

Contact: vitess@googlegroups.com  **·**  
Announcements  **·**  
© 2017 Vitess powered by Google Inc
